# The Negative Association between Breastfeeding Duration and Infant Febrile Seizure: A Retrospective Case-Control Study

**DOI:** 10.3390/ijerph19095495

**Published:** 2022-05-01

**Authors:** Hsi-Yu Chen, Yu-Hsun Chang, Dah-Ching Ding

**Affiliations:** 1 Department of Medicine, Hualien Tzu Chi Hospital, Buddhist Tzu Chi Foundation, Hualien 970, Taiwan; 103311145@gms.tcu.edu.tw; 2 Department of Pediatrics, Hualien Tzu Chi Hospital, Buddhist Tzu Chi Foundation, Hualien 970, Taiwan; 3 Department of Obstetrics and Gynecology, Hualien Tzu Chi Hospital, Buddhist Tzu Chi Medical Foundation, Tzu Chi University, Hualien 970, Taiwan; 4 Institute of Medical Sciences, Tzu Chi University, Hualien 970, Taiwan

**Keywords:** breastfeeding, febrile seizures, duration, low birth weight, preterm labor

## Abstract

Two to five percent of infants and children experience febrile seizures (FS). Breastfeeding is beneficial to the health of mothers and children. Nevertheless, the benefits of breastfeeding in reducing FS remain unclear; thus, the present study aimed to evaluate this association. The case group was selected from 2010 to 2019, and the selected population was children younger than 5 years (i.e., children born from 2005–2019). The control group was selected from newborn infants at our hospital born between 2005 and 2019. Finally, 55 children with FS and 110 children in the control group were recruited. The results show longer breastfeeding duration is associated with an increased risk of FS (adjusted odds ratio: 1.06, 95% confidence interval: 1.01–1.11, *p* = 0.028). When comparing cases of FS with the control group, the percentage of inclusive breastfeeding over 12 months (32.7% vs. 9.1%, *p* = 0.017) and longer duration of exclusive breastfeeding were higher (10.86 ± 11.82 vs. 5.40 ± 7.17 months, *p* < 0.001). However, the comparison of the prevalence of FS between the different breastfeeding duration groups did not reach statistical significance. In conclusion, our study showed that a longer breastfeeding duration was associated with a higher risk of FS. Future large-scale studies evaluating the association between breastfeeding duration and febrile seizures are needed.

## 1. Introduction

Febrile seizure (FS) is a seizure accompanied by a fever without central nervous system infection [1]. It is the most common form of seizure in early childhood, and usually occurs between 3 months and 5 years of age. Two to five percent of infants and children experienced FS in the United States and Western Europe. The incidence was even higher in other parts of the world [2]. The exact cause of FS is unknown but is thought to be caused by both genetic and environmental factors [3]. Except for the influence of genetics, risk factors including a family history of FS, peak body temperature during the illness, low birth weight, and in utero growth retardation have been reported. The effects of prenatal exposure to smoking and alcohol are still under debate [2,4,5].

The importance of breastfeeding has also been recognized. The World Health Organization and the American Academy of Pediatrics recommends at least six months of exclusive breastfeeding [6]. Breastfeeding has also been reported to be beneficial to the health of mothers and children [7], and has a protective effect in infants with infectious [8,9,10] and metabolic diseases [11]. Furthermore, there is growing evidence of an association between breastfeeding and improved neurocognitive development in children [6,12].

There is little evidence showing the association between breastfeeding and FS, and the effect of breastfeeding duration has scarcely been discussed [4,13]. Thus, the aim of this study was to determine the association between breastfeeding and FS, and to discuss the influence of different breastfeeding durations.

## 2. Materials and Methods

### 2.1. Study Design

The study was approved by the Research Ethics Committee of Hualien Tzu Chi Hospital (IRB110-109-278-B). This was a single-institutional retrospective case-control study conducted at the Hualien Tzu Chi Hospital. The subjects included 55 cases of FS and 110 matched control cases of the same gender and similar age.

### 2.2. Sample Selection

#### 2.2.1. Selection of Cases

We first searched electronic medical records and identified cases of children below five years of age who were diagnosed with FS during admission, in the emergency room, or in the outpatient department of pediatrics between 1 January 2010 and 31 December 2019. The subjects were 255 cases registered at Hualien (designated by an ID number starting with “U”). By reviewing the charts of each case, those with missing information, those with a diagnosis of epilepsy or preterm labor, and those with brain injury were excluded. In total, 182 patients were included. We obtained information on feeding patterns and missing maternal background information through phone calls to the parents of each included case. The number of included cases decreased to 89 after excluding those who did not pick up the phone or refused to provide information.

#### 2.2.2. Matched Sample Design and Selection of Control Group Cases

**Figure 1:**
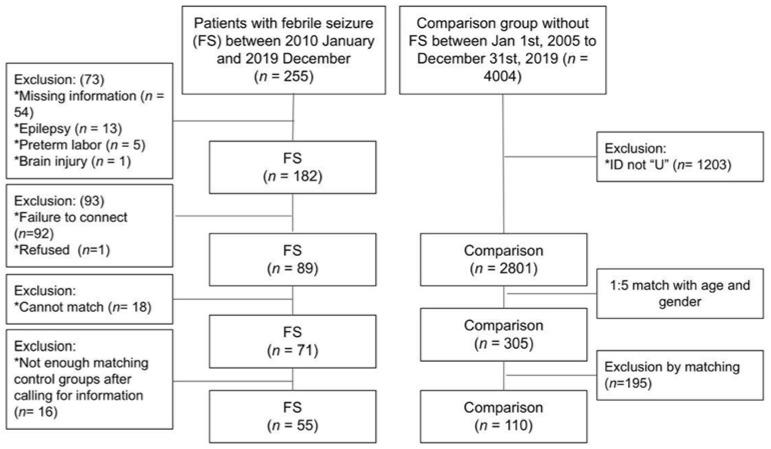
Study flow chart.

We aimed to select controls with other children of the same gender and the same birth month and year as the cases in the study. As the earliest cases may have been 5 years old in 2010, the earliest year of birth was 2005. Thus, for the control group, it was necessary to include infants born from 2005–2019. For the comparison group, we identified 4004 infants discharged from our hospital nursery from 1 January 2005 to 31 December 2019. The comparison cases were selected from a larger time frame to allow for the inclusion of a larger control group, so a case to control ratio of 1:5 could be achieved. The comparison cases were selected from a time frame which encompassed the year of birth for some of the earlier cases of FS to allow for accurate case matching. Similar to the case group, 2801 cases with ID numbers starting with “U” were included. We then matched the 89 cases and control groups with the same gender and birth month and year with a ratio of one to five. Eighteen cases of FS did not match the control group. The remaining 71 cases of FS and 355 control cases were included. We screened the charts of control cases and called the parents of these cases for feeding patterns and missing maternal background information simultaneously. Patients with missing information, a history of febrile seizures, epilepsy, brain injury, or preterm labor through chart review were excluded. Those who did not pick up their phone or refused to provide information through phone calls were also excluded. After iterating through the matching pairs, the subjects included 55 cases of FS and 110 control cases (Figure 1).

### 2.3. Data Collection

#### 2.3.1. Basic Characteristics and Maternal Background Information

Information of basic characteristics including age and gender of the cases were obtained simultaneously when we searched for the cases. Information on birth weight and the number of siblings was obtained from the charts. Maternal background information, including gestational age, mode of delivery (cesarean section or normal spontaneous delivery), multiple births, pregnancy complications (gestational bleeding, preeclampsia, gestational diabetes mellitus), and family history of epilepsy, were mostly collected from the charts, while information on alcohol intake, smoking, anticonvulsant usage during pregnancy, maternal history of epilepsy, and maternal age were obtained through phone calls. Information that was not recorded in the charts was collected through phone calls.

#### 2.3.2. Assessment of Feeding Patterns

Breastfeeding patterns were recorded using phone calls. We called each case and collected information including the duration of breastfeeding (both inclusive and exclusive), and the duration of infant formula feeding until 1 year of age after fully informing them of the purpose of the research. We chose the groups of lactation duration to match the time of children’s routine visits to the clinics. Because of this, the records in the charts were more accurate and reliable.

### 2.4. Statistical Analysis

We planned a study of matched sets of cases and controls with 2 matched control(s) per case according to age and gender. We assumed that the probability of exposure among controls was 50%, the correlation coefficient for exposure between matched cases and controls was 0.2, and the true odds ratio for disease in exposed subjects relative to unexposed subjects was 3. After setting Type I error as 5% and power as 80%, we estimated that at least 51 case patients with 102 matched controls were needed according to the R package epiR [14].

In order to compare the baseline characteristics, maternal background, and breastfeeding details between the case and control groups, an independent t-test was used for continuous variables, whereas the chi-squared test or Fisher’s exact test was performed for categorical variables. Next, we compared the characteristics among different groups of breastfeeding duration by performing one-way ANOVA and a post hoc test (Bonferroni’s correction) for continuous variables and chi-squared test or Fisher’s exact test for categorical variables. Finally, we performed conditional logistic regression to compare the characteristics of patients with febrile seizures with those of matched control groups. SPSS software (version 24, IBM, New York, NY, USA) was used for analysis. Statistical significance was set at *p* < 0.05.

## 3. Results

### 3.1. Demographics

Table 1 shows the demographic characteristics of the two groups. The percentage of low birth weight was higher in the FS cases (16.4% vs. 2.7%, *p* = 0.003). The gestational age was also smaller in the FS cases (38.26 ± 1.33 vs. 38.89 ± 1.03 weeks, *p* = 0.001). Maternal complications (bleeding, alcohol consumption, history of epilepsy, and family history of FS) were also higher in infants with FS. Breastfeeding duration was also higher in the FS cases (10.86 ± 11.82 vs. 5.40 ± 7.17 months, *p* < 0.001).

### 3.2. Characteristics of Different Breastfeeding Duration Groups

Next, we analyzed the characteristics of groups categorized by breastfeeding duration, including durations that were shorter than a month (*n* = 46), between a month and six months (*n* = 46), and longer than six months (*n* = 73). Regarding sex, in the breastfeeding 1–6 months group, the percentage of male babies reached 80.4% (the post hoc test showed that the male ratio in group 2 was significantly higher than group 1 and 3) (Table 2). Although the prevalence of FS and low birth weight between groups did not reach a significant difference, the children who were breastfed for more than 6 months had a higher prevalence of FS (39.7%) and low birth weight (8.2%) compared to those who were breastfed between 1 and 6 months (28.3%; 4.3%) (Table 2). The other characteristics were not statistically significant among the groups. The post hoc test also showed that the duration of breastfeeding was significantly shorter in group 1 than group 2 and 3. Nevertheless, the groups were divided by duration of breastfeeding.

### 3.3. FS risk Factors Analysis

The conditional logistic regression analysis results of the characteristics among children with FS, which were compared with matched control groups, suggest that children with longer gestational age (odds ratio [OR]: 0.64; 95% confidence interval [CI]: 0.47–0.87) have a lower risk of FS. The risk of FS was higher among those with low birth weight (OR: 8.29; 95% CI: 1.78–38.69), with a family history of febrile seizure (OR: 5.16; 95% CI: 1.63–16.31), longer breastfeeding duration (OR: 1.06; 95% CI: 1.02–1.10), and those whose mothers had alcohol intake during pregnancy (OR: 5.99; 95% CI: 1.21–29.73). After adjusting the variables, the association between FS and longer breastfeeding duration (adjusted OR: 1.06; 95% CI: 1.01–1.11) remained (Table 3).

## 4. Discussion

The main result of our study shows that a longer breastfeeding duration is associated with an increased risk of FS. In addition, when comparing cases of FS with the control group, the percentage of inclusive breastfeeding over 12 months and longer duration of exclusive breastfeeding were higher. However, the comparison of the incidence of FS between the different breastfeeding duration groups (BF < 1 M, 1–6 M, >6 M) did not reach statistical significance (*p* = 0.3).

There are few previous studies on the association between breastfeeding and FS, and they indicated the positive role of breastfeeding in FS. A large-scale birth cohort study in Japan showed a negative relationship between breastfeeding and FS [4]. Of 84,321 children included in the analysis, 6264 (7.4%) were reported to have an FS episode at least once in the first three years of life [4]. A longer duration of breastfeeding tended to decrease the incidence of FS. Additionally, male sex and increased fever episodes were associated with FS. Another case-control study in China also concluded that children with exclusive breastfeeding reduced the incidence of FS [13]. Three hundred and thirty-six patients with FS and 3336 febrile children matched with age and gender were enrolled as the control group. Children with exclusive breastfeeding had a lower risk of FS compared with formula feeding (OR: 0.504, 95% CI: 0.303–0.841). Partial breastfeeding did not reach a significant role in decreasing FS. In contrast, our study showed that an extended period of breastfeeding increased the risk of FS. A possible reason for such a result might be related to factors we did not assess, such as education level or family income.

Low birth weight has been reported to be a risk factor for FS in children. A higher susceptibility to infection and fever episodes is considered to be the cause [15,16,17]. The previous study showed the 5-year cumulative incidence of FS increased with decreasing birth weight [<1500 g; 5.42% (95% CI: 4.98–5.88% vs. 3000–4000 g; 3.53% (95% CI: 3.50–3.56%)] [15]. Another populational study showed no statistical significance between FS and low birth weight birth at late preterm birth (OR: 1.08, 95% CI: 0.97–1.20) [18]. A longitudinal analysis of the national sample cohort from 2002 to 2013 also revealed no association between FS and low birth weight (OR: 0.96, 95% CI: 0.65–1.41) [19]. Our results are consistent with those of previous studies showing a higher percentage of low birth weight among children with FS. Regarding the association between low birth weight and breastfeeding duration, our study showed no significant difference, but it showed an increased percentage in the group that had breastfeeding for >6 months. We also found similar results when comparing the percentage of FS with different breastfeeding durations.

Family history has been reported to be an important factor for both recurrent FS and epilepsy [4,20,21]. A study by Aslan recruited 82 patients with FS and showed that a family history of epilepsy or FS was the main risk factor for FS [21]. Kumar et al. reported in a one-year follow-up study of 528 children aged 6 months to 5 years that a family history of FS was associated with the risk of FS (OR: 3.72, 95% CI: 2.27–6.10) [22]. Tarhani et al. recruited 77 patients with FS who showed that a family history of seizures was associated with FS (*p* = 0.036) [23]. Our study showed not only an increased prevalence of FS among those with a family history of FS and maternal family history of epilepsy but also detected that a family history of FS is a risk factor for FS. A shorter breastfeeding duration was found among mothers with a family history of epilepsy in a previous study [4]. However, no association was found between breastfeeding duration and a family history of FS or epilepsy in our study.

Vestergaard et al. reported no impact on the risk of FS with prenatal exposure to low to moderate levels of alcohol and also found a weak association between maternal smoking and FS [24]. Later, Vahidnia et al. found that children born of mothers who both smoke and drink may have a higher risk of FS, but they found no harmful effects independently [25]. In our study, we observed an increased risk of FS among mothers with alcohol consumption during pregnancy, but no risk was found among those who smoked during pregnancy. As we did not assess the effect of prenatal smoking and alcohol consumption simultaneously, future studies are needed to confirm these findings.

The sociocultural characteristics of mothers may have influenced FS incidence and breastfeeding duration. Indeed, mothers with more education and those with higher family income are associated with a longer duration of breastfeeding and a decreased incidence of FS [4]. Our study did not investigate the sociocultural characteristics as the relevant data was not present in our database.

The strengths of our study are as follows. First, this is the first study to evaluate the association between breastfeeding and FS in Taiwan. We further analyzed the effect of different breastfeeding durations on the prevalence of FS. Furthermore, we included diverse confounding factors such as prenatal environmental factors, maternal history, and birth history.

However, our study has some limitations. There were only few cases of complications during pregnancy, maternal history of epilepsy, or anticonvulsant use during pregnancy due to the small sample size. Thus, we were not able to analyze the association between FS and these factors and we could not adjust for these variables. In addition, recall bias regarding breastfeeding duration was inevitable, as the study was designed as a retrospective case-control study. Moreover, socioeconomic factors that might have an effect on the occurrence of FS were not included in our study. Additionally, half of the initially included cases could not be contacted by phone. Thus, the exclusion number was high, which may have constituted a further bias and limitation of our study. Finally, our study included a small number of cases, which may have been a further limitation of our study. In the future, prospective and large-sample-size studies are required to provide a more thorough and evidential conclusion.

## 5. Conclusions

In conclusion, our study showed that a longer breastfeeding duration was associated with a higher risk of FS. This result is different from previous findings that reported the protective effect of breastfeeding on FS. Future large-scale studies evaluating the association between breastfeeding duration, low birth weight, and FS are warranted.

## Figures and Tables

**Table 1:** Demographics (*n* = 165).

Item	Control (without FS)	Case (with FS)	Total	*p* Value
*N*	110	55	165	
Basic characteristics				
Age	2.16 ± 1.06	2.16 ± 1.06	2.16 ± 1.06	1.000
Gender				1.000
Girls	44(40.0%)	22(40.0%)	66(40.0%)	
Boys	66(60.0%)	33(60.0%)	99(60.0%)	
Birth weight (g)	3112.77 ± 391.54	3013.22 ± 473.90	3079.99 ± 421.59	0.156
Low birth weight (<2500 g) (%)	3(2.7%)	9(16.4%)	12(7.3%)	0.003 *
Maternal background information				
Gestational age	38.89 ± 1.03	38.26 ± 1.33	38.69 ± 1.17	0.001 *
Mode of delivery				0.335
NSD	80(72.7%)	36(65.5%)	116(70.3%)	
CS	30(27.3%)	19(34.5%)	49(29.7%)	
Maternal age	30.72 ± 4.64	29.76 ± 5.58	30.4 ± 4.97	0.247
Complications at pregnancy (%) *n* = 160	0(0.0%)	5(10.0%)	5(3.1%)	0.003 *
Gestational bleeding (%) *n* = 164	0(0.0%)	5(9.3%)	5(3.0%)	0.003 *
Preeclampsia (%)	0(0.0%)	0(0.0%)	0(0.0%)	1.000
DM (% )	1(0.9%)	2(3.6%)	3(1.8%)	0.258
Smoking during pregnancy (%)	1(0.9%)	3(5.5%)	4(2.4%)	0.108
Alcohol intake during pregnancy (%)	2(1.8%)	6(10.9%)	8(4.8%)	0.017 *
Anticonvulsant use during pregnancy (%)	0(0.0%)	1(1.8%)	1(0.6%)	0.333
Maternal history of epilepsy (%)	0(0.0%)	3(5.5%)	3(1.8%)	0.036 *
Family history of FS (%)	5(4.5%)	11(20.0%)	16(9.7%)	0.002 *
Breastfeeding details				
Feeding patterns				0.017 *
No Breastfeeding	32(29.1%)	6(10.9%)	38(23.0%)	
<1 month	1(0.9%)	7(12.7%)	8(4.8%)	
1–2 month	11(10.0%)	7(12.7%)	18(10.9%)	
3–4 month	16(14.5%)	3(5.5%)	19(11.5%)	
5–6 month	6(5.5%)	3(5.5%)	9(5.5%)	
7–<12 month	25(22.7%)	7(12.7%)	32(19.4%)	
12 month	9(8.2%)	4(7.3%)	13(7.9%)	
>12 month	10(9.1%)	18(32.7%)	28(17.0%)	
Duration	5.40 ± 7.17	10.86 ± 11.82	7.22 ± 9.32	<0.001 *

DM, diabetes mellitus; FS, febrile seizure; NSD: normal spontaneous delivery; CS: cesarean section. Data are presented as *n* or mean ± standard deviation. * *p*-value < 0.05 was considered statistically significant after test.

**Table 2:** Comparison of characteristics among different breastfeeding groups (*n* = 165).

Item	BF < 1 M (Group 1)	BF 1–6 M (Group 2)	BF > 6 M (Group 3)	Total	*p* Value	Post-Hoc
*N*	46	46	73	165		
Basic characteristics						
Age	2.04 ± 0.91	2.26 ± 1.02	2.18 ± 1.16	2.16 ± 1.06	0.610	
FS (%)	13(28.3%)	13(28.3%)	29(39.7%)	55(33.3%)	0.300	
Gender					0.004 *	1 < 2, 3 < 2
Girls	21(45.7%)	9(19.6%)	36(49.3%)	66(40.0%)		
Boys	25(54.3%)	37(80.4%)	37(50.7%)	99(60.0%)		
Birth weight (g)	3070.60 ± 388.58	3067.59 ± 309.68	3093.6 ± 499.83	3079.99 ± 421.59	0.934	
Low birth weight (<2500 g) (%)	4(8.7%)	2(4.3%)	6(8.2%)	12(7.3%)	0.747	
Maternal background information						
Gestational age	38.76 ± 1.17	38.57 ± 1.09	38.72 ± 1.22	38.69 ± 1.17	0.700	
Mode of delivery					0.278	
NSD	36(78.3%)	29(63.0%)	51(69.9%)	116(70.3%)		
CS	10(21.7%)	17(37.0%)	22(30.1%)	49(29.7%)		
Maternal age	31.43 ± 4.40	30.07 ± 4.80	29.96 ± 5.37	30.40 ± 4.97	0.252	
Multiple birth (%)	0(0.0%)	1(2.2%)	0(0.0%)	1(0.6%)	0.558	
Complications at pregnancy (%) *n* = 160	1(2.2%)	0(0.0%)	4(5.7%)	5(3.1%)	0.278	
Gestational bleeding (%) *n* = 164	0(0.0%)	0(0.0%)	5(6.8%)	5(3.0%)	0.075	
Preeclampsia (%)	0(0.0%)	0(0.0%)	0(0.0%)	0(0.0%)	1.000	
DM (%)	2(4.3%)	0(0.0%)	1(1.4%)	3(1.8%)	0.461	
Smoking during pregnancy (%)	2(4.3%)	0(0.0%)	2(2.7%)	4(2.4%)	0.388	
Alcohol intake during pregnancy (%)	3(6.5%)	1(2.2%)	4(5.5%)	8(4.8%)	0.728	
Anticonvulsant use during pregnancy (%)	1(2.2%)	0(0.0%)	0(0.0%)	1(0.6%)	0.558	
Maternal history of epilepsy (%)	2(4.3%)	0(0.0%)	1(1.4%)	3(1.8%)	0.461	
Family history of FS (%)	4(8.7%)	2(4.3%)	10(13.7%)	16(9.7%)	0.260	
Breastfeeding details						
Duration	0.06 ± 0.18	2.41 ± 1.42	14.75 ± 9.57	7.22 ± 9.32	<0.001 *	1 < 2 < 3

BF, breastfeeding; DM, diabetes mellitus; FS, febrile seizure. Data are presented as *n* or mean ± standard deviation. * *p*-value < 0.05 was considered statistically significant.

**Table 3:** Conditional logistic regression analysis characteristics among patients of FS, compared with matched controls.

Characteristic	Crude			Adjusted		
	OR	95% CI	*p*-Value	OR	95% CI	*p*-Value
Maternal age	0.96	(0.90, 1.03)	0.236	0.99	(0.90, 1.09)	0.841
Gestational age	0.64	(0.47, 0.87)	0.004 *	0.73	(0.50, 1.06)	0.096
Diabetes mellitus (Yes vs. No)	4.00	(0.36, 44.11)	0.258	1.58	(0.10, 25.02)	0.745
Smoking during pregnancy (Yes vs. No)	6.00	(0.62, 57.68)	0.121	1.25	(0.02, 68.54)	0.914
Alcohol intake during pregnancy (Yes vs. No)	5.99	(1.21, 29.73)	0.028 *	15.12	(0.84, 273.60)	0.066
Family history of FS (Yes vs. No)	5.16	(1.63, 16.31)	0.005 *	3.08	(0.73, 12.94)	0.124
Low birth weight (<2500 g) (Yes vs. No)	8.29	(1.78, 38.69)	0.007 *	3.63	(0.65, 20.21)	0.142
Breast feeding duration	1.06	(1.02, 1.10)	0.001 *	1.06	(1.01, 1.11)	0.028 *

OR, odds ratio; CI, confidence interval. * *p*-value < 0.05 was considered statistically significant after test. Case and control groups were matched with age and gender. The conditional logistic regression model was adjusted for the basic characteristics and maternal background information listed in Table 1.

## Data Availability

All relevant data are reported in the article.
